# From Sweet to Sour: SGLT-2-Inhibitor-Induced Euglycemic Diabetic Ketoacidosis

**DOI:** 10.3390/jpm14070665

**Published:** 2024-06-21

**Authors:** Andrijana Koceva, Nika Aleksandra Kravos Tramšek

**Affiliations:** 1 Department of Endocrinology and Diabetology, University Medical Center Maribor, Ljubljanska Ulica 5, 2000 Maribor, Slovenia; andrijana.koceva@ukc-mb.si; 2 Faculty of Medicine, University of Maribor, Taborska ulica 8, 2000 Maribor, Slovenia

**Keywords:** SGLT-2 inhibitors, euglycemic diabetic ketoacidosis, mechanisms, precipitating factors, prevention

## Abstract

Sodium–glucose cotransporter 2 (SGLT-2) inhibitors are highly selective, effective, and generally well-tolerated antihyperglycemic agents targeting the SGLT-2 transmembrane protein. Despite being primarily registered for diabetes treatment, due to their cardiorenal protective properties, SGLT-2 inhibitors caused a paradigm shift in the treatment of other diseases on the cardiorenal spectrum, becoming a fundamental part of heart failure and chronic kidney disease management. With their rapidly increasing use, there are also increased reports of a rare, often under-recognised and potentially deadly side effect, SGLT-2-inhibitor-induced euglycemic diabetic ketoacidosis (EDKA). The primary pathophysiological process behind its multifactorial aetiology comprises glucosuria and osmotic diuresis, which produce a significant carbohydrate deficit, leading to an increase in the glucagon–insulin ratio, thus resulting in accelerated ketogenesis. Although EDKA has a similar clinical presentation as diabetic ketoacidosis (DKA), the absence of the high glucose levels typically expected for DKA and the presence of urine ketone reabsorption contribute to a significant delay in its recognition and timely diagnosis. Given the broad use of SGLT-2 inhibitors, increased awareness, early recognition, and prompt identification of precipitating factors are essential. In this narrative review, we comprehensively explore the pathophysiological mechanisms of SGLT-2-inhibitor-induced EDKA, analyse its clinical manifestation, and identify the most common triggers for its development. We also discuss EDKA management and preventive strategies.

## 1. Introduction

Sodium–glucose cotransporter 2 inhibitors (SGLT-2 inhibitors) are highly selective, effective, and generally well-tolerated anti-hyperglycaemic agents approved for treating adults with type 2 diabetes mellitus. They offer the convenience of once-daily oral administration and carry a low inherent risk of hypoglycaemia. As a result of their insulin-independent mechanism of action, SGLT-2 inhibitors can be used for monotherapy and as a component of combination therapy with other antidiabetic agents with complementary modes of action to improve glycaemic control in patients with type 2 diabetes [1,2]. They are effective, safe, and well-tolerated in frail, elderly people [3].

Despite being primarily used for the treatment of diabetes, the indications of SGLT-2 inhibitors for other diseases on the cardiorenal spectrum have rapidly expanded, even in patients without diabetes. They have demonstrated a significant reduction in the risk of heart failure irrespective of pre-existing cardiovascular disease [4]. At the moment, SGLT-2 inhibitors such as empagliflozin and dapagliflozin are also approved for the treatment of chronic heart failure, regardless of left ventricular ejection fraction, as well as for reducing cardiovascular events and the risk of kidney disease progression in patients with chronic kidney disease [1,5,6,7,8,9]. SGLT-2 inhibitors increase diuresis and reduce the use of loop diuretics during hospitalisation without having a negative effect on renal function [10,11]. They reduce albuminuria and improve tubular reabsorption, which in turn reduces hypertension and hyperfiltration, thereby preserving tubular function and the glomerular filtration rate [12,13].

Diabetic ketoacidosis (DKA) is a life-threatening complication of diabetes that occurs more often in type 1 diabetes and less often in type 2 diabetes [14]. DKA development occurs due to absolute or relative insulin deficiency in combination with increased levels of counterregulatory hormones such as glucagon, catecholamines, cortisol, or growth hormone [15]. DKA is characterised by the triad of hyperglycaemia (>13.9 mmol/L), metabolic acidosis with an increased anion gap (pH < 7.3, serum bicarbonate < 15 mmol/L, and anion gap > 10–12 mmol/L), and increased blood and urine ketone levels [16]. Euglycemic diabetic ketoacidosis (EDKA) was first described in 1973 by Munro et al. [3,4,5]. It is defined by the presence of hyperglycaemia or relative euglycemia (blood glucose concentration < 14 mmol/L), elevated ketone levels (ketonemia or significant ketonuria), and metabolic acidosis with an elevated anion gap (bicarbonate < 18 mmol/L and/or venous blood pH < 7.3; anion gap > 10) [17].

In May 2015, the US Food and Drug Administration (FDA) published a warning about the potential increased risk of SGLT-2-inhibitor-induced DKA based on 20 cases of DKA, and by June 2015, the European Medicines Agency also reported 147 cases of SGLT-2-inhibitor-induced DKA [18]. A meta-analysis of randomised controlled trials later showed a 2.58 to 5.29 times higher risk of DKA compared to the control group. Treatment with SGLT-2 inhibitors increases the risk of DKA in both type 1 and type 2 diabetes, and the risk increase in in type 2 is around 7-fold higher [18]. Despite the use of SGLT-2 inhibitors, other EDKA-precipitating factors include infection, exertion or physical stress, decreased caloric intake or prolonged fasting, anorexia, persistent vomiting, dehydration, gastroparesis, insulin pump failure, insulin dose reduction/omission, acute pancreatitis, (bariatric) surgery, alcohol use disorder, ketogenic diet, glycogen storage disease, chronic liver disease, newly diagnosed diabetes mellitus, and pregnancy [14,16,19,20].

## 2. The Aim

This article aims to explore the pathophysiological mechanisms of SGLT-2-inhibitor-induced EDKA, analyse its clinical manifestations, and compare its clinical manifestations and treatment strategies with those of DKA, which can aid treating physicians in the earlier recognition and more efficient management of this complication. We aim to identify the most common triggers for its development and explore preventive strategies.

## 3. Materials and Methods

We performed a literature search using PubMed and identified articles published between 2014 and 2024 about EDKA development in SGLT-2 inhibitor users. The keywords used included, but were not limited to, “empagliflozin” OR “dapagliflozin” OR “sodium-glucose cotransporter-2” OR “SGLT-2 inhibitors” AND “euglycemic diabetic ketoacidosis” OR “EDKA”. PubMed yielded 217 articles. A literature search of articles cited in the identified publications was also conducted. We evaluated case reports and case series; cross-sectional, retrospective studies; systematic reviews; meta-analyses; and randomised controlled trials. Published guidelines were also reviewed. The literature search was restricted to articles and studies written in English, focusing on EDKA secondary to SGLT-2 inhibitor use. Based on the findings, in this narrative review, we comprehensively explore the pathophysiological mechanisms of SGLT-2-inhibitor-induced EDKA, analyse its clinical manifestation, and identify the most common triggers for its development. Management and preventive strategies are also discussed in detail.

## 4. Mechanisms of SGLT-2-Inhibitor-Induced EDKA

The main fuel for the brain is glucose, and in times of low cellular glucose availability, ketone bodies are another source. Ketogenesis is the process of ketone formation in the liver, where acetoacetate and 3-beta (β) hydroxybutyrate are formed from acetyl-CoA [21]. Both ketones are released from the liver in a protonated form, so depending on the volume and buffering capacity of the blood, their release lowers the pH of the blood. The level of ketones in the blood determines the equilibrium between ketogenesis and ketolysis. Ketoacidosis occurs when the balance is disturbed and the rate of hepatic ketone formation exceeds the rate of ketone utilisation. Both processes are controlled by insulin and glucagon. Insulin inhibits lipolysis and hepatic ketogenesis as well as increases ketone utilisation and oxidation in the peripheral tissue, therefore ultimately lowering blood ketone levels. Glucagon, on the other hand, increases hepatic ketogenesis by stimulating hormone-sensitive lipase to mobilise free fatty acids from adipose tissues [21].

SGLT-2 inhibitors increase the risk of ketoacidosis in several ways; central to these processes is an increase in the glucagon–insulin ratio. SGLT-2 inhibitors inhibit sodium–glucose cotransporter 2 in the proximal tubule, which is responsible for reabsorbing 80–90% of filtered glucose. Reduced reabsorption and glucosuria result in a decrease in blood glucose levels [22,23]. As blood glucose decreases, a carbohydrate deficit develops, resulting in a reduced insulin dose requirement in insulin-treated patients and reduced endogenous insulin secretion from the pancreas. As insulin concentrations decrease, its anti-lipolytic activity also decreases, increasing the production of free fatty acids, which are then converted into ketone bodies by beta-oxidation in the liver [16,23,24]. Insulin also increases the activity of acetyl-CoA carboxylase, resulting in the production of malonyl-CoA, which is a potent inhibitor of carnitine-palmitoyl transferase I (CPT-I). Since CPT-I stimulates the transport of free fatty acids into the mitochondria, thereby increasing their beta-oxidation by lowering insulin levels indirectly via reduced CPT-I inhibition, an additional increase in ketogenesis occurs [16,24]. By lowering blood glucose levels, SGLT-2 inhibitors indirectly stimulate glucagon secretion, which inhibits acetyl-CoA carboxylase activity and increases CPT-I activity in the liver, further contributing to increased ketogenesis [16,18,24]. Some studies indicated that SGLT-2 is also expressed in human pancreatic alpha cells and contributes to glucose sensing in islets. In this way, SGLT-2 inhibition could also have a direct positive stimulatory effect on alpha cells, further explaining the increase in glucagon levels with SGLT-2 inhibitor treatment. However, the latter has not been sufficiently investigated, and evidence in this area is inconsistent [15,21,23,24] as other studies stated that SGLT-2 inhibition may also stimulate glucagon secretion indirectly by acting on SGLT-2 receptors in the brain, promoting a catecholamine increase, which further stimulates alpha cell glucagon secretion [16,25]. This combination of decreased insulin and increased glucagon secretion leads to a higher glucagon–insulin ratio which favours ketogenesis. Potential interventions to modulate the glucagon–insulin ratio can include insulin therapy, which supresses ketogenesis and promote tissue glucose uptake, as well as regular carbohydrate intake and avoiding low-carbohydrate or ketogenic diets as both can increase glucagon levels and therefore ketone body production [26]. Additionally, this increased ratio can also be modulated using glucagon receptor antagonists, which were explored recently in a randomised, double-blind, placebo-controlled crossover trial by Boeder et al. in which they compared SGLT-2 inhibitor therapy in patients with type 1 diabetes with the combination of SGLT-2 inhibitors and glucagon receptor antagonists. This study showed improved glucose control and diminished ketogenesis in patients taking combination therapy [27].

Additionally, SGLT-2 inhibitors increase ketone body reabsorption by decreasing sodium reabsorption in the proximal tubule, which increases sodium concentration in the distal tubule, resulting in an electrochemical gradient favouring acetoacetate and β-hydroxybutyrate reabsorption. SGLT-2 inhibitor users may therefore have significant ketoacidosis without ketonuria [23]. The renal response to acidosis and the rate of ketoacid clearance are also reduced, leading to marked ketonemia and the classic high anion gap in ketoacidosis [16]. Reduced sodium reabsorption in the proximal tubule leads to a relative excess of ATP in the kidney, which shifts the kidney away from metabolic pathways that typically produce ATP, including ammoniogenesis and ketone body oxidation, further worsening metabolic acidosis [23].

Treatment with SGLT-2 inhibitors also increases osmotic diuresis and decreases intravascular volume. This leads to direct β1-adrenergic receptor activation, which further increases glucagon secretion [28]. In the case of illness, nausea, an inability to consume fluids, or starvation, more significant volume loss and dehydration may occur, which also accelerates the development of ketoacidosis [20,21]. An additional factor can also be an inappropriate or excessive reduction in the insulin dose when prescribing SGLT-2 inhibitors to insulin-dependent patients, resulting in additional insulin deficiency and leading to enhanced lipolysis and hepatic ketogenesis [23].

**Figure 1:**
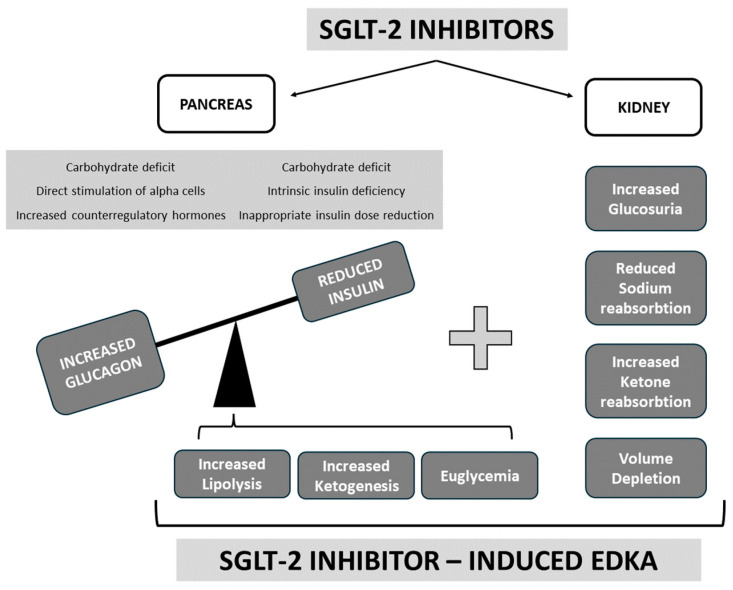
Potential mechanisms of SGLT-2-inhibitor-induced EDKA development.

In summary, SGLT-2 inhibitors cause a carbohydrate deficit, leading to lower insulin levels, and may directly or indirectly stimulate glucagon secretion, increasing the glucagon–insulin ratio. This presents with increased lipolysis and ketogenesis. On the other hand, by acting on the kidney, SGLT-2 inhibitors cause increased glucosuria, decreased sodium reabsorption, and increased ketone reabsorption and volume depletion, additionally contributing to faster EDKA development in the right clinical setting. Figure 1 summarises the potential mechanisms of SGLT-2-inhibitor-induced EDKA development.

## 5. Clinical Manifestations and Diagnosis of SGLT-2-Inhibitor-Induced EDKA

The risk of EDKA development is higher in patients with diabetes mellitus type 1, diabetes mellitus type 2 with severe insulin deficiency, patients with a low body mass index, and patients with low glycogen stores [14]. EDKA can present at any time after the initiation of an SGLT-2 inhibitor, although some suggest that it may occur more commonly in the first two months [14,17]. An analysis of data from the FDA’s Adverse Event Reporting System showed that SGLT-2-inhibitor-induced DKA develops in patients with a wide range of ages, body weights, and treatment durations [29]. This shows that caution should be exercised in all patients receiving SGLT-2 inhibitor treatment.

The symptoms of EDKA are similar to those of DKA, but the lack of hyperglycaemia often leads to a delay in diagnosis. Patients with EDKA complain of nausea and vomiting and may have abdominal pain, shortness of breath, and fatigue. Tachycardia, hypotension, dry mucous membranes, and poor skin turgor may also be present upon examination. Depending on the degree of acidosis, Kussmaul’s breathing and varying degrees of altered consciousness may also occur [14,17,23,30]. In a review of 72 cases of SGLT-2-inhibitor-induced EDKA, Menghoum et al. showed that the most common symptoms were nausea in 48%, abdominal pain in 38%, and vomiting in 36%, while the most common signs were tachypnoea in 34%, tachycardia in 30%, dehydration in 14%, and altered consciousness in 10% [28]. In a recent report, Almazrouei et al. retrospectively analysed the clinical and biochemical characteristics of 55 patients with type 2 diabetes mellitus admitted with DKA. They showed that EDKA was present in 56.3% of SGLT-2 inhibitor users compared to 2.6% of non-users. Despite lower glucose levels, the SGLT-2 inhibitor users exhibited higher sodium levels, lower systolic blood pressure, and higher urea levels, indicating worse hypovolemia. Additionally, acute kidney injury was more common in the SGLT-2 inhibitor users and, compared to non-users, the SGLT-2 inhibitor users were five times more likely to have a prolonged hospital stay (≥14 days) [31]. A recent meta-summary of 169 clinical cases of SGLT-2-inhibitor-induced EDKA by Juneja et al. revealed that patients most commonly reported gastrointestinal symptoms, with nausea and vomiting reported by 65.1% and abdominal pain by 37.3%, followed by respiratory symptoms such as breathlessness in 30.8% [32].

To confirm the diagnosis of EDKA, a blood glucose value of less than 13.9 mmol/L is determined, and an arterial or venous blood gas analysis is performed. The anion gap is calculated to confirm metabolic acidosis with a high anion gap, and blood levels of β-hydroxybutyrate and acetoacetate are determined to confirm ketonemia and/or urine ketones to confirm ketonuria [17,23,30,33]. The most common ketone body measurement recommended is β-hydroxybutyrate, for which a level of >3 mmol/L is considered significant. If serum ketone measurements are unavailable, urine ketones can also be measured, though this can lead to false negative results because SGLT-2 inhibitors increase urinary ketone reabsorption [14,17,34]. As EDKA is a diagnosis of exclusion, other causes of metabolic acidosis with an increased anion gap, such as lactic acidosis, ethanol, methanol, or ethylene glycol poisoning, salicylate, tricyclic antidepressant or paracetamol poisoning, and renal failure, should permanently be excluded [35]. Differentially, ketoacidosis due to starvation or alcoholic ketoacidosis is also diagnostically relevant [17,22]. In the meta-summary by Juneja et al., the median blood glucose level at diagnosis was 184.5 mg/dl (10.3 mmol/L), and in most cases, there was severe metabolic acidosis with a median pH of 7.14, a serum bicarbonate level of 8.6 mmol/L, and a high anionic gap of 22.5 mmol/L. The median lactate levels in this meta-summary were only 1.3 mmol/L [32], showing that hyperlactatemia is uncommon. Similar results were shown by Menghoum et al., with the mean glycemia level being 15.69 mmol/L, an HbA1c of 8.9 ± 2.2%, a pH of 7.2 ± 0.17, serum bicarbonate levels of 10.5 ± 5.8 mmol/L, and lactate levels of 1.7 ± 1.3 mmol/L despite the substantial use of metformin in 88% of patients studied, again confirming that lactic acidosis is an unlikely driver of the metabolic acidosis seen in SGLT-2-inhibitor-induced EDKA [28].

To summarise, EDKA can occur at any time after SGLT-2 inhibitor initiation. Most commonly, it manifests with gastrointestinal symptoms such as nausea, abdominal pain, or vomiting, followed by respiratory symptoms and altered consciousness. Laboratory tests show metabolic acidosis with a high anion gap due to ketonemia with or without ketonuria. Although clinical and laboratory test outcomes are similar to those in classic DKA, patients lack indicative hyperglycaemia but can have higher sodium and urea values, indicating worse hypovolemia.

## 6. Treatment of SGLT-2-Inhibitor-Induced EDKA

The treatment of EDKA is similar to the treatment of hyperglycaemic DKA. Blood glucose should be checked every hour, and electrolytes and blood pH should be checked every 2–4 h. The first steps in the treatment of EDKA include the withdrawal of the SGLT-2 inhibitor and intensive fluid replacement with 1–2 L of crystalloid fluids in the first 2 h of treatment. A 0.9% sodium chloride solution is usually given at a rate of 500–1000 mL/h intravenously; after that, a decision is made to reduce the infusion rate to 200–500 mL/h and, depending on the serum sodium level, to change the infusion solution to 0.45% sodium chloride [14,17,26,36]. The next step is a correction of any hypokalaemia via intravenous potassium replacement and the introduction of a continuous insulin infusion. Insulin is usually injected intravenously at a continuous infusion rate of 0.05–0.1 units/kg/h, which lasts until ketoacidosis resolves or the patient can eat normally, as required, until the complete resolution of acidosis and the normalisation of serum bicarbonate occur. After the resolution of acidosis, defined as pH > 7.30 and serum bicarbonate > 18 mEq/L, subcutaneous insulin can be initiated. Insulin infusion should be continued for 1–2 h after subcutaneous insulin administration [14,26]. In a retrospective analysis, Sell et al. analysed and compared the outcomes of patients with EDKA to those with DKA. In this analysis, only 14% of EDKA was related to SGLT-2 inhibitors. Patients with EDKA had milder ketoacidosis at presentation, a shorter total hospital stay, a shorter mean time of parenteral insulin infusion without a difference in the average time to the first long-acting subcutaneous insulin application, and a serum bicarbonate elevation > 18 mmol/L. Also, rates of hypoglycaemia in patients with EDKA were increased compared to patients with DKA, leading to the conclusion that when treating patients with EDKA, adjustments to the standard DKA treatment protocol which involve using a slower rate of insulin infusion and higher dextrose concentrations while during insulin infusion are needed [37]. Insulin primarily prevents further ketone formation, lowers the blood glucose concentration, and allows a more rapid correction of electrolyte imbalances. Since blood glucose levels in EDKA are usually lower than 11–14 mmol/L, an infusion of 5–10% glucose is often used alongside insulin infusion in order to prevent hypoglycaemia. Bicarbonate replacement is only necessary in exceptional cases of life-threatening acidosis with a pH ≤ 6.9. Treatment should be continued until ketoacidosis has resolved or the pH, anionic gap, and ketone levels have normalised [14,15,16,17,23,38].

The mortality of patients with DKA is <1% in the United States and similar in Europe (from 1 to 5%), but it can be more than 10% in countries with limited acute care resources. If patients have two or more episodes of DKA, the mortality hazard ratio is 2.8–4.5 times higher. Compared with hyperglycaemic hyperosmolar syndrome, the rates are even higher (5–16%) [39]. Although data on the mortality and morbidity of SGLT-2-inhibitor-induced EDKA are lacking, according to a meta-summary of 169 case reports, the overall mortality rate of SGLT-2-inhibitor-induced EDKA is only 2.4% [32].

To summarise, the crucial parts of SGLT-2-inhibitor-induced EDKA treatment are SGLT-2 discontinuation and fluid and insulin replacement. Although EDKA treatment is similar to the treatment of hyperglycaemic DKA, due to the lower glucose values, 5–10% glucose replacement is often needed alongside the insulin infusion. Bicarbonate replacement is rarely needed. Treatment is continued until pH, anion gap, and ketone levels normalise.

## 7. Trigger Identification and Prevention Strategies

As SGLT-2 inhibitor-induced EDKA usually results from a combination of different triggers, one should also focus on identifying and treating all the triggering factors that lead to EDKA development. In a study performed by Ata et al. following patients with type 2 diabetes treated with SGLT-2 inhibitors, infection was the most common precipitating factor for both EDKA and classic hyperglycaemic DKA development, followed by insulin non-compliance, pancreatitis, and surgery. Although likely due to the study’s retrospective nature, an identifiable precipitating factor was not found in 51% of cases [40]. In the meta-summary of 169 cases of SGLT-2-inhibitor-induced EDKA carried out by Juneja et al., an identifiable triggering factor was found in 78.7% of cases; several patients reported multiple triggering factors, and the most common precipitating factor was acute severe infection followed by s perioperative period [32]. In the review of 72 cases of SGLT-2-inhibitor-induced EDKA performed by Menghoum et al., prolonged fasting, surgical intervention, infection, and recent dose reduction or cessation of insulin therapy were the most common triggers [28].

Before initiating an SGLT-2 inhibitor, a detailed history should be taken to assess risk factors that might increase the patient’s susceptibility to ketoacidosis and to identify patients at an increased risk of developing DKA, such as patients with reduced residual insulin secretion or type 3 diabetes (e.g., patients with latent autoimmune diabetes in adults (LADA) or a history of pancreatitis) [26,28]. Some studies suggest screening patients for LADA before prescribing SGLT-2 inhibitors [26]. Patients should also be educated about the importance of adequate hydration and carbohydrate intake when initiating an SGLT-2 inhibitor [21,33]. In the case of DKA development in a patient with presumed type 2 diabetes, consideration should be given to the accuracy of the diagnosis as Hamblin et al. showed that 22% of patients thought to have type 2 diabetes ended up being reclassified as having type 1 diabetes or LADA in both SGLT-2 users and non-SGLT-2 users [41].

SGLT-2 inhibitors lower the threshold for ketoacidosis development in times of infection, illness, stress, and low carbohydrate intake [42]. Patients are advised against the excessive consumption of alcoholic beverages or very low-carbohydrate or ketogenic diets, both of which can worsen and accelerate the development of ketoacidosis [15,26,43]. When a drug is introduced in an insulin-treated patient, modifications to the insulin therapy dose are often performed, but the discontinuation or excessive reduction in insulin therapy doses should be avoided, and patients should also be instructed on the correct modification of insulin therapy doses in the event of disease [26,30]. Since EDKA can develop if significant insulin reductions are made at the time of SGLT-2 inhibitor treatment initiation, this should be an important consideration for hospitalised patients as there are often significant changes or reductions made to patients’ insulin regimens by clinicians during hospital stays [44]. A retrospective, multicentre, controlled cohort study by Hamblin et al. showed that patients using SGLT-2 inhibitors were more likely to develop DKA during inpatient stays compared to non-users. DKA developed in 38% of patients treated with SGLT-2 inhibitors, with the median blood glucose value being 13.5 mmol/L compared to 2% of patients using other medical treatments for type 2 diabetes, with the median glucose value here being 25.4 mmol/L [41].

Although the American Association of Clinical Endocrinologists and the American College of Endocrinology first recommended stopping SGLT-2 inhibitors at least 24 h before elective surgery back in 2016 [34], as the elimination half-life of SGLT-2 inhibitors ranges from 11 to 13 h and the pharmacodynamic effects of SGLT-2 inhibitors may persist for several days after discontinuation, it may be appropriate to stop SGLT-2 inhibitors approximately three days (or five half-lives) before major surgery [23,34].

According to the latest FDA Drug Safety Communication, revised in 2022, to lower the risk of DKA after surgery, a three-day discontinuation of canagliflozin, dapagliflozin, and empagliflozin and at least a four-day discontinuation of ertugliflozin should be considered before scheduled surgery [45]. Other international associations recommend temporary discontinuation three days before elective surgery and immediately in case of emergency surgery, acute illness, fever, or dehydration [23,26,33]. The Australian Diabetes Society also advised temporarily discontinuing SGLT-2 inhibitors for at least three days before surgery and procedures requiring one or more days of hospital stay and/or requiring “bowel preparation,” such as colonoscopy. If the SGLT-2 inhibitor is part of a fixed-dose combination, this will lead to the withdrawal of two glucose-lowering drugs unless the second drug is continued separately. This temporary cessation may require increasing other glucose-lowering drugs [46]. For shorter procedures (including gastroscopy), SGLT-2 inhibitors can be stopped only on the day of the procedure. Fasting before and after the procedure should be minimised. If the SGLT-2 inhibitor has not been temporarily stopped for 3 days before the procedure or if it has been taken on the day of surgery or the day of the procedure, the appropriate course of action should be determined based on the urgency of the procedure and surgical factors combined with patient comorbidity, HbA1c, blood ketone level, and base excess [46].

EDKA, therefore, is more likely to develop during or following emergency surgery due to the inability to anticipate and prepare for the temporary discontinuation of SGLT-2 inhibitors. To decrease the probability of EDKA development in these cases, the perioperative administration of insulin and glucose infusions should be considered [47,48].

Special consideration should also be given to patients undergoing bariatric surgery. Here, apart from anticipated surgical stress, patients often have additional risk factors for DKA development, such as a period of a ketogenic or very low-carbohydrate diet before surgery and a prolonged low-carbohydrate diet after surgery [49]. Patients are often placed on a low-carbohydrate and high-protein diet 1–2 weeks before surgery in order to enhance laparoscopic operative field visualisation by decreasing hepatic glycogen stores and hepatic mass. In these cases, some recommend stopping SGLT inhibitors up to two weeks before surgery. The risk of ketoacidosis is also significant in the postoperative period, mostly due to the combination of rapid weight loss, the presence of nausea and vomiting, as well as an inadequate or low-carbohydrate diet [23,30]. DKA presentation in patients can show as late as six weeks post bariatric surgery [30].

Previous guidelines on DKA recognition advise ketone testing on sick days or whenever glucose deterioration occurs. Due to the lack of typical indicative hyperglycaemia in EDKA, this may not be adequate for patients taking SGLT-2 inhibitors [50]. Here, patient education is of particular importance as patients need to be aware of the possibility of developing DKA while having lower blood glucose levels and should be educated on the potential triggers of EDKA and receive a lower threshold for ketone testing. Urine ketone measurements in patients taking SGLT-2 inhibitors may be misleading, which is why blood ketone testing is recommended in these cases [50]. A continuous ketone-monitoring technology that detects interstitial fluid β-hydroxybutyrate levels may also be a promising innovative solution for enabling the safer use of SGLT-2 inhibitors in high-risk patients or aiding in early EDKA detection during hospitalisation, perioperative periods, and during transitional care following hospital discharge [51,52].

So far, there are no known implications of the long-term use of SGLT-2 inhibitors, and patients with pre-existing risk factors for EDKA do not need prevention strategies other than those listed above. Clinicians should be aware that people who have SGLT-2 inhibitors prescribed for diabetes are more likely to develop EDKA than those using SGLT-2 inhibitors for heart failure or chronic kidney disease treatment, as mentioned above.

In summary, SGLT-2 inhibitors lower the threshold for ketoacidosis development in times of infection, illness, perioperative stress, and low carbohydrate intake, which is why several preventive methods should be considered when treating patients with SGLT-2 inhibitors. SGLT-2 inhibitor users should avoid excessive alcohol consumption and a low-carbohydrate or ketogenic diet. When ill, SGLT-2 inhibitors should be discontinued without the discontinuation of insulin treatment and, if available, blood ketone testing should be performed regularly despite a lack of hyperglycaemia. SGLT-2 inhibitors should be discontinued three (canagliflozin, dapagliflozin, and empagliflozin) or four (ertugliflozin) days before surgery, and if urgent surgery is needed, considering a perioperative intravenous insulin and glucose infusion may be beneficial. As no specific biomarkers are available for recognising patients with an increased risk for SGLT-2-inhibitor-induced EDKA, educating patients as well as clinicians on common EDKA-triggering factors and preventive measures is essential.

## 8. Future Directions

Given their broad use, future research should conduct large-scale prospective studies to identify specific patient characteristics that increase the risk of SGLT-2-inhibitor-induced EDKA as predictive models and biomarkers are lacking and focus on creating and validating tools for its early detection. It would be interesting to compare the incidence and clinical manifestation of EDKA in patients receiving SGLT-2 inhibitors for diabetes as opposed to other indications. Also, the long-term outcomes for patients who experienced SGLT-2-inhibitor-induced EDKA are not well-established, including the impact on diabetes management, quality of life, and potential recurrent episodes. Preventive strategies for EDKA in the perioperative setting are well established, but since infections are common EDKA triggers in users of SGLT-2 inhibitor, precise guidelines for discontinuing and re-starting SGLT-2 inhibitors during periods of illness are needed. Specific guidelines on the use and discontinuation of SGLT-2 inhibitors in the inpatient setting are also missing. Lastly, there are limited data on whether different SGLT-2 inhibitors vary in the risk of inducing EDKA. Addressing these gaps in future research will be beneficial for improving diabetes outcomes and ensuring patient safety.

## 9. Conclusions

In recent years, SGLT-2 inhibitors have proven to be highly effective drugs. Due to their mechanism of action, the prescription indications have expanded to include diseases other than diabetes. According to the evidence to date, these are safe drugs, but caution should be exercised for using SGLT-2 inhibitor in the presence of other EDKA precipitating factors. EDKA is a rare but potentially deadly side effect of SGLT-2 inhibitor therapy, often with a multifactorial aetiology. Given the undeniable cardiovascular and renal benefits and the expected widespread use of SGLT-2 inhibitors for the benefit of patients, we need to ensure greater awareness of the prompt recognition and initiation of EDKA treatment as well as the identification of its triggers for the appropriate prevention of EDKA.

## Data Availability

No new data were created or analyzed in this study. Data sharing is not applicable to this article.
